# Open source bioimage informatics for cell biology

**DOI:** 10.1016/j.tcb.2009.08.007

**Published:** 2009-11

**Authors:** Jason R. Swedlow, Kevin W. Eliceiri

**Affiliations:** 1 Wellcome Trust Centre for Gene Regulation and Expression, College of Life Sciences, University of Dundee, Dundee, Scotland DD1 5EH, UK; 2 Laboratory for Optical and Computational Instrumentation, Departments of Molecular Biology and Biomedical Engineering, Graduate School, University of Wisconsin at Madison, Madison, WI 53706, USA

## Abstract

Significant technical advances in imaging, molecular biology and genomics have fueled a revolution in cell biology, in that the molecular and structural processes of the cell are now visualized and measured routinely. Driving much of this recent development has been the advent of computational tools for the acquisition, visualization, analysis and dissemination of these datasets. These tools collectively make up a new subfield of computational biology called bioimage informatics, which is facilitated by open source approaches. We discuss why open source tools for image informatics in cell biology are needed, some of the key general attributes of what make an open source imaging application successful, and point to opportunities for further operability that should greatly accelerate future cell biology discovery.

## Bioimage Informatics as a discovery tool in cell biology

Imaging is used as a tool for discovery throughout basic life science, and biomedical and clinical research. In these domains, advances in light and electron microscopy have transformed biological discovery, enabling visualization of mechanism and dynamics across scales of nanometers to millimeters and picoseconds to many days. Fluorescent protein (FP)-tagged fusions can be used as reporters of biomolecular interactions in cultured living cells [1], and the same reporter can reveal the localization and growth of a tumor in a living animal [2,3]. In short, the last 20 years have provided us with a wealth of sophisticated biological reporters and image data acquisition tools for biomedical research. Many of these imaging and instrumentation developments have been driven by partnerships between academic laboratories that invent and prototype new technology and commercial entities that develop and market them as commercial products. This development and delivery pipeline of commercial imaging instrumentation and software has been quite successful, having delivered the laser scanning confocal [4,5], spinning disc confocal [6,7], wide-field deconvolution [8,9] and multiphoton microscopes [10] that are engines of discovery in cell and developmental biology.

All of these methodologies produce complex, multi-dimensional data sets that must be transformed into reduced representations that scientists can manipulate, analyze, share with colleagues, and ultimately understand. Despite the diversity of applications of imaging in biology, there are common unifying challenges such as displaying a multi-gigabyte time-lapse movie on a laptop screen, or identifying, tracking, and measuring the objects in that movie and presenting the resulting measurements in a graph that reveals the mechanisms that drive their movements. These requirements have spawned the new field of bioimage informatics [11], which aims to deliver tools for data visualization, management, storage, and analysis. While still a relatively young field, bioimage informatics has already had a major impact in cell biology particularly in the area of quantitative cell imaging where advanced feature recognition, segmentation, annotation and data mining approaches are used regularly [12–20].

Almost all commercially provided image acquisition systems include software tools that provide sophisticated image visualization and analysis functions for the images recorded by the instrument they control. However, in recent years, many non-commercial projects have appeared, almost always based in research laboratories that require functionality not available in commercial products. Here, we discuss the application of bioimage informatics in cell biology and focus specifically on the development of open source solutions for bioimage informatics that have emerged over the last few years.

## What are the informatics challenges in quantitative cell biology imaging?

Given the rapid development in image acquisition systems in the last 20 years, it is worth considering why a corresponding rapid development of informatics tools has occurred only recently. Certainly, one of the barriers to providing universal tools for bioimage informatics is the diversity of data structures and experimental applications that produce imaging data. In optical microscopy alone there are a substantial number of different types of imaging modalities and, indeed, a method like fluorescence microscopy encapsulates a huge and rapidly growing field of image acquisition approaches [21]. Informatics tools that support this range of methods must be capable of capturing the raw data (the individual pixels) and the metadata around the acquisition methodology itself, including instrument settings, exposure details etc. This diversity of data structures makes delivering common informatics solutions difficult, and this complexity is multiplied by the large number of commercial imaging systems that use individually specified, and often proprietary, file formats for data storage. Our current estimates are that there are approximately 80 proprietary file formats for optical microscopy alone (and not including other common imaging techniques) that must be supported by any bioimage informatics tool that aims to provide a generalizable solution. In short, the lack of standardized access to data makes the generation of informatics tools quite difficult.

A deeper challenge resides in each individual laboratory that uses imaging as part of its experimental repertoire. The sheer size of the raw data sets and the rate of production mean that individual researchers can easily generate many tens of gigabytes of data per day. This means that large laboratories or departmental imaging facilities generate many hundreds of gigabytes to terabytes per week, and are now enterprise-level data production facilities. However, the expertise for developing enterprise software tools or even simply running the hardware necessary for this scale of data management and analysis rarely exists in individual laboratories. In short, the sophisticated systems and development expertise that are used to deliver genomics databases and applications are required in individual imaging laboratories and facilities. The delivery of tools that provide access to a broad range of data types, manage and analyze large sets of data, and help run the systems that store and process this data is the challenge that bioimage informatics seeks to address.

## Why are open source approaches essential?

A critical development in the field of bioimage informatics has been the introduction of many open source projects in the last few years [11,22–30]. These projects range from being open source distributions where the code is available but new development is not specifically encouraged, to open development projects that are community-driven projects that actively encourage the help and participation of projects for the support and addition of new features. Therefore, before we proceed, it is worth considering what constitutes open source and open development efforts and why they are valuable or even necessary for bioimage informatics.

Open source software is a well-established movement with strong paradigms in many very successful projects such as Linux (http://www.linuxfoundation.org/), Java (http://java.sun.com/), MySQL (http://www.mysql.com/products/database/), and Apache (http://www.apache.org/). A fundamental tenet of open source software projects is that the copyright holder (usually the software developer or his/her employer) determines the software license, which defines how the software is distributed and what end-users may do with the software. For open source software, the original source code is made available under the terms of this license. An open source license usually allows end-users to use the software for any purpose, make changes to the software source code or link their own software to it and, if they desire, distribute those ‘derivative works. However, the software license also defines under what terms and license derivative works may be distributed. Table 1 gives some examples of commonly used open source software licenses and summarizes their terms. For any users or developers, these details are important and must be understood given the great implications for development and deployment.

The ability to see and make changes to the work of another developer is a critical component of open source software. The attractive aspect of this approach for science is that users and developers can directly see, evaluate, and use another's work (really, their intellectual property) and, if necessary, build upon it. This is a key and often overlooked part of open source software. Successful open source software development projects are dynamic, evolving enterprises allowing input, feedback, and often contributions from their community.

This evolving, adaptable aspect makes open source software particularly useful for scientific discovery and, more specifically, for the rapidly evolving and diverse set of imaging applications used in biological research. Commercial and closed source applications have certainly supported many significant advances in imaging. However, an essential part of bioimaging data analysis is the ability to easily try new methodology and approaches or even to combine existing ones to generate a derivative result based on the combination of two approaches. Open source approaches make this possible. As such, there is a natural fit between open source software and the process of scientific discovery. In addition, a consequence of the growth of the open source community is a de facto establishment of standardized documentation methods (http://java.sun.com/j2se/javadoc/) and software specifications (http://java.sun.com/products/ejb/docs.html). These specifications ensure that developers can understand and use each other's code and, most importantly, that two independent software packages can use a specified, common interface. This software ‘interoperability’, enforced by the community either formally or informally, is a general hallmark of open source software, and perhaps one of its most underappreciated strengths. Because standardization is so well established in the open source community, open source software has a critical role in providing the specifications and tools for common file formats or common interfaces that enable two otherwise incompatible packages to communicate their input and output data to one another. This type of interoperability is critical to support the rapidly evolving needs of bioimage informatics. For all these reasons, many of the recent developments in bioimage informatics are based on an open source foundation.

Recently, a subclass of open source project know as ‘open development’ has been defined (http://www.oss-watch.ac.uk/resources/odm.xml). Open development projects take the open source concepts and add a significant role for the community in the development process. In truth, community interaction and feedback was a component of most initial open source projects, but as open source projects have expanded, not all have included efforts to engage and respond to their user community. Community interaction and support is expensive, it takes precious developer time and often requires the use of forums, mailing lists, and other resources to manage the interactions with the project's community. However, open source, and open development approaches in particular, have proven to be particularly attractive for funding agencies supporting biomedical research. They provide a way to measure the success of the project, by providing measures of uptake and participation. As the community grows around an open development project, it provides a measure of protection for the research investment and sustainability of the software past the duration of the initial award. Many agencies are now requiring that applicants have a software sharing plan in their grant application and, if an open source approach is not possible, justify this decision. In our opinion, the value for the developers, the community and the funding investment will be maximized if open development models are also followed.

## Open source tools for data acquisition, visualization, analysis, and dissemination

It is beyond the scope of this article to provide a comprehensive review of all available open source tools in image informatics and features and applications. Many other papers [20,27–36] have reviewed particular applications in depth. Instead, we list representative ones (Table 2) to help illustrate the current impact and future potential of open source tools in biological imaging.

## Supporting open source software

Open source software drives further innovation by allowing the free exchange of code and algorithms. Commercial applications are largely driven by market demand for a specific function or feature, so proprietary software has to be economically viable and thus must have feature limitations, code access restrictions and design parameters focused on a particular user base. Open source software complements these commercial packages and allows for new scientific ventures where a desired feature or code addition may not be commercially viable to develop.

Any open project must be viable, it must deliver valuable products to its community, and it must be sustainable and have a strategy for long-term funding. In academic science, many projects receive grant funding to initiate their work, but it is common for software development to require more than three years to achieve a fully developed product that can be distributed and used by the community. Sustaining these efforts exclusively through grants is possible, but requires convincing demonstration of the software's utility, and must accept the reality that continued funding is subject to variations in availability of funding and the priorities of funding organizations. As they mature, most open source software efforts develop a non-profit foundation (e.g. Apache Software Foundation, http://www.apache.org) or a commercial arm (e.g. http://www.kitware.com and http://glencoesoftware.com) that can directly access funding from user communities through licensing and customization fees that support the targeted customer base and help finance additional code development and maintenance for the open source package. However, there are still few examples of this maturation in scientific software. An important question for the scientific community is what priority funding agencies should place on the continued funding of software development tools for its use. If continued funding is to be considered, the application and reviewing processes will need to be modified to properly capture and assess the value of these projects. In our opinion, in exchange for periodic review and consideration for sustained funding, publicly funded scientific software projects should be required to follow open development models, where engagement and support for the community is required. This can occur only if funding for support and community engagement is available, and if career development and evaluation include publication record and delivery of useful tools to and engagement with the community.

In comparing open source and commercial software products, one of the biggest differences is support for the software itself. In general, commercial software packages are supported with instructions, manuals, and direct user support, and this is a key advantage of using commercial software. The cost of such support is either included in the original purchase price or paid for by purchase of a software maintenance agreement. Covering the costs of user support is difficult for open source projects because there is no corresponding fee structure to cover such support costs and, often, the academic grants that fund open source projects cover only the innovative research components and do not support the personnel or infrastructure needed. This is gradually changing with funding agencies and scientists alike realizing the importance of producing innovative and feature-rich code but ensuring that it is well supported and maintained. There are well-established standards and tools in the open source community for support, mailing lists, user forums, screencast demos, and wiki-based user documentation, that all contribute to making software successful. Within our own Open Microscopy Environment Consortium (http://openmicroscopy.org), we use project management tools such as Subversion (http://subversion.tigris.org/) to manage our source code repository, Trac (http://trac.edgewall.org/) for all project management and issue and revision tracking, Jabber (http://www.jabber.org) for real-time communication, Hudson (https://hudson.dev.java.net/) for continuous integration, Plone for managing our web site (http://plone.org/), and PHPBB for running our user forums (http://www.phpbb.com/). In addition to these tools, we hold annual user meetings to assess progress and define roadmaps for future works. We participate as presenters or exhibitors in large meetings of the community in order to capture as much feedback as possible. These tools and activities help support and engage a very broad user and developer community and are an important part of ensuring community wide adoption, but installing, running, and maintaining these tools, as well as answering queries and moderating discussions, requires time and resources (both people and money). Many successful open source packages have shown the importance of transforming the conventional user base into an additional support mechanism where the user community interacts with the original developers and with each other for support and new code developments. Users and developers that are new to the project are often supported by the community, and not just the main development team. This transformation takes some time and investment because it results from releasing useful software and investing a moderate amount of resources in support. However, we strongly advocate that direct funding of support personnel and tools be made available for research-based open source software development. In our experience, many of our academic colleagues hesitate to release their software because of the burden of supporting use of their software, thus preventing the synergies that should occur within the scientific community.

While many of these arguments are in support of open source software for scientific research in general, there are specific advantages for biological imaging. Bioimaging is inherently interdisciplinary and covers a wide range of technical and biological applications. Given this great heterogeneity of its technology and applications, bioimaging needs the open exchange of techniques and principles for further innovation. There has always been a rich tradition of this from the physical instrumentation side of bioimaging development; many current emerging imaging technologies were first developed in other fields (e.g., adaptive optics [37] was first developed in astronomy). Open source software development builds upon this collaborative instrumentation approach to allow for further innovation by sharing specific algorithms or leveraging specific code for data acquisition, visualization or data sharing.

## Partnering commercial and open source efforts

Most of the imaging systems in biological laboratories are commercially developed and provided, and thus driven by commercially licensed, closed software. These powerful tools are the workhorses of modern biological research. There are many examples of companies using open source specifications to increase the functionality and value of their products, including major vendors such as Red Hat and IBM. In addition, the expertise and know-how in commercial companies is valuable, and open source projects are often aided by commercial partners working as supporters and as active developers. We therefore strongly advocate partnerships between commercial providers and open source software projects. With the appropriate licensing models, companies can be actively involved in open source imaging software for the benefit of all. Micromanager (http://www.micro-manager.org/) and the Open Microscopy Environment [20,28,38] are two prominent examples of this where commercial providers have made significant contributions and played active roles in software development. Academic and commercial partnerships are vital to the long-term success and innovation in bioimage informatics, just as this same arrangement has facilitated new innovation in imaging hardware development.

## Summary

The rapid innovation in imaging technology for biomolecules, cells, and tissues requires a parallel development in software tools for managing visualizing and analyzing image data. Open source software has an important role in this development, as open code development and sharing enable rapid exchange and experimentation with new tools and ideas. As open source software tools become more sophisticated, funding mechanisms that enable laboratories to provide long-term support to a broad user and developer community must be made a priority by funding bodies. The open community is very interactive and evaluates performance based on merit; i.e. good software is used by its target audience. Thus, further funding can be tied to feedback from and uptake by the community. Our experience is that academic software should follow an open development model, even if this approach deviates from the standard models used for academic research. It is important that any funded open source program be managed efficiently and integrate previous efforts and community specifications. Finally, the community must understand that a development project does ‘develop’; it grows, matures, and ultimately, if properly run and integrated with its user community, delivers useful tools. The community's comments and feedback during this growth is critical. This process is slow and iterative, but the paradigms are well established and can be used to deliver successful tools and ultimately new discoveries.

## Figures and Tables

**Table 1:** List of features of common open source licenses.

Open source licenses	Website	Key features
Public domain	http://www.copyright.gov/title17/92chap1.htmlhttp://en.wikipedia.org/wiki/Public_domain	Software distributed in this way has no license or any restrictions based on its use, modification, or distribution. The software is not subject to any copyright protection
Apache license	http://www.apache.org/licenses/	Allows for commercial use and allows for modified code to be distributed freely under any license. Considered one of the most liability-resistant open source licenses due to clear language in the license
BSD license	http://www.linfo.org/bsdlicense.html	Allows for unrestricted distribution as long as copyright and warranty notices are included
GNU general public license (GPL)	http://www.gnu.org/licenses/gpl-3.0.txt	All derived works must use the same license. Notably less permissive than BSD
GNU lesser General public audience (LGPL)	http://www.gnu.org/copyleft/lesser.html	More permissive version of GPL that allows programs that just link with LGPL code to not be LGPL.
Mozilla public license	http://www.mozilla.org/MPL/MPL-1.1.html	Hybrid of GPL and BSD requiring all used and modified code to stay under license. Has several key additions, including allowing executables to use different licenses and explicit clause on patent rights
Common development and distribution license	http://www.sun.com/cddl/	Free license developed by Sun but considered incompatible with GPL. Based on the Mozilla license, it has several key additions to ease commercial use including patentability adoption and notice of use.
Common public license	http://www.opensource.org/licenses/cpl1.0.php	Must make source code available but allows for proprietary programs to use without being same license type
MIT license (also called X11 license)	http://www.opensource.org/licenses/mit-license.php	GPL compatible license that is permissive allowing for proprietary software use

**Table 2:** Representative survey of open source bioimage tools

Software tool	Main feature set	License type	Website
ImageJ	Image processing	Public domain	http://rsbweb.nih.gov/ij/
OME	Database and image informatics	GPL	www.openmicroscopy.org
Bio-Formats	Metadata interchange library	GPL	www.loci.wisc.edu/ome/formats.html
CellProfiler	Automated identification of features in cells	GPL	www.cellprofiler.org/
VisBio	Multidimensional image analysis	GPL	www.loci.wisc.edu/visbio
Bisque	Database system for semantic analysis	GPL	www.bioimage.ucsb.edu/
PSLID	Subcellular localization data model	GPL	murphylab.web.cmu.edu/services/PSLID/
Micromanager	Microscopy control	BSD	www.micro-manager.org/
ITK	Set of extensive tools for image analysis, including segmentation and registration	BSD	www.itk.org/
FARSIGHT	Toolkit for associative image analysis	BSD	www.farsight-toolkit.org
Osirix	Multidimensional medical image viewer	GPL	www.osirix-viewer.com/
BioImageXD	Python package that leverages ITK for image processing and volume rendering	GPL	www.bioimagexd.net/
